# A large pediatric retroperitoneal benign lipoma in a 2-year-old female: A case report and literature review

**DOI:** 10.1097/MD.0000000000046360

**Published:** 2026-05-12

**Authors:** Batool Fatima, Neha Rubab, Muhammad Umer Farooq Mujahid, Sakarie Mustafe Hidig

**Affiliations:** a Department of Pediatric Surgery, Mayo Hospital, Lahore, Pakistan; b Liaquat National Hospital and Medical College, Karachi, Pakistan; c University of Health Sciences, Lahore, Pakistan; d Research Center, Hargeisa Group Hospital, Hargeisa, Somalia.

**Keywords:** benign retroperitoneal lipomatous tumors, case report, children, lipoblastoma, retroperitoneal lipoma

## Abstract

**Rationale:**

Lipomatous tumors are rare in the pediatric population. Lipomatous tumors may develop at any location throughout the body; however, they are highly uncommon in the retroperitoneal area. Lipomatous tumors represent the predominant category of soft-tissue neoplasms. Only 3 cases of pediatric retroperitoneal lipoma have been reported since 1980. We report a benign retroperitoneal lipomatous tumor and the literature compilation of benign retroperitoneal lesions.

**Patient concerns:**

A 2-year-old South Asian female presenting with 13 months of increasing abdominal distention and intermittent fever was referred to our hospital. The child had no trauma or gastrointestinal symptoms like vomiting, diarrhea, or constipation.

**Diagnoses:**

An abdominal ultrasound showed a large, echogenic mid-abdomen tumor. The tumor obscured the bowel without calcification or fat necrosis. A color Doppler scan indicated no blood flow in the lesion. The computed tomography scan of the neck, chest, and abdomen with IV contrast showed a well-circumscribed, non-enhancing, heterogeneous hypodense mass from the left hypochondrium to the pelvis on the left abdominal region. The mass measured 18.4 × 14.8 × 13.6 cm, and its Hounsfield units ranged from −66 to −90, indicating adipose tissue. Histopathology showed a benign lipoma made of mature fat cells and thinsultory fibrils with no malignancy, cellular atypia, or necrosis.

**Interventions:**

The patient had surgical resection via laparotomy, achieving complete resection with negative margins.

**Outcomes:**

The patient was discharged on the 4th day after the procedure, after an uneventful recovery. After 6 weeks, the child was asymptomatic in follow-up.

**Lessons:**

The long-term prognosis of the retroperitoneal lipoma among children remains inadequately characterized relative to adults due to a small number of cases. Current literature suggests magnetic resonance imaging, computed tomography scan, and FISH are the most suitable diagnostic options. Extended follow-up is essential in pediatric patients.

## 1. Introduction

A lipoma is a benign tumor composed of mature adipocytes. Retroperitoneal lipomas (RPL) are uncommon neoplasms that generally manifest in adults between the ages of 40 and 60 years, with only a few instances documented in pediatric populations.^[1]^ Primary retroperitoneal tumors constitute merely 0.2 percent of total body tumors.^[2]^ These benign neoplasms originate from mesenchymal tissue cells and can occur in nearly any location within the body. Nevertheless, they are predominantly located in the subcutaneous tissue of the extremities and thorax. Retroperitoneal benign lipomas are pretty rare, accounting for just about 2.9% of all primary retroperitoneal tumors.^[2]^

The RPL can be discussed within the domain of soft-tissue pathology because it was historically classified as well-differentiated liposarcoma. The relevance of the issue in clinical settings requires that the surgical and pathological communities accept RPL as a specific diagnostic category to reduce unnecessary overtreatment and prevent unnecessary surveillance.^[3]^ In recent years, due to the widespread use of fluorescence in situ hybridization, which enables the elimination of CDK4/murine double minute-2 amplification – the molecular hallmark of well-differentiated – the diagnostic reliability of RPL has improved significantly.^[3,4]^

Because essential systems are not very common and loose connective tissue is common, most retroperitoneal lipomas do not cause any symptoms until they attain a significant size. As a result, they are frequently identified inadvertently or during gastrointestinal assessments.^[5]^

## 2. Case presentation

**Figure 1.**
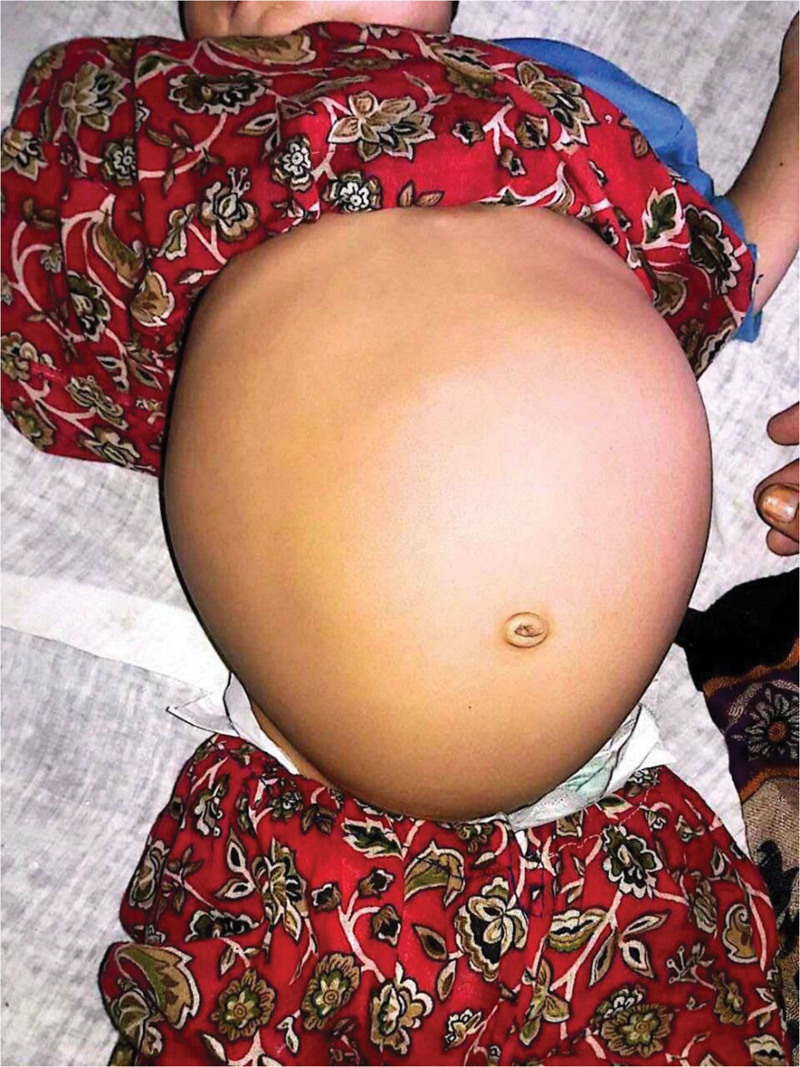
Abdominal distention due to retroperitoneal lipoma.

A 2-year-old female was admitted to our hospital with a 13-month history of progressive abdominal distension, and on intermittent occasions, there were episodes of fever. The patient did not have any trauma or gastrointestinal dysfunction, including vomiting, diarrhea, or constipation. She had no other medical history and no notable family history of such conditions. The physical examination disclosed a substantial, hard, non-tender mass in the belly extending to the left hypochondrium and the pelvic region, as shown in Figure 1. No indications of systemic illness, such as weight loss or peritonitis, were observed. The timeline of patient clinical occurrences along with management interventions is shown in Table 1.

**Table 1:** Chronology regarding patient clinical occurrences along with management interventions.

Event	Timeline
Onset of abdominal distention	13 mo before
Referral to surgeon	1 mo before
CT, ultrasound & biopsy executed	18 d before
Surgical procedure conducted	0th day
Discharged from hospital	4th day
Follow-up (first)	6 wk post-op
Follow-up (second)	8 post-op months (not occurred)

CT = computed tomography.

Laboratory tests were routine. The total blood count and inflammatory markers did not indicate any infection or cancer. The doctors addressed financial and cultural diagnostic challenges, and this case report included a single patient; neither any intervention was contemplated for the purpose of the study, nor was patient management affected. Thus, no ethical approval was required according to the journal policy. The father of the patient gave his written informed consent. The whole process was based on the principles of the Declaration of Helsinki. An abdominal ultrasonography revealed a large, highly echogenic neoplasm in the middle part of the abdomen, measuring approximately 9.0 × 9.2 cm. The lesion obscured bowel morphologies without showing calculi. Color-Doppler examination showed no vascular supply in the mass, which was indicative of malignancy, and it was followed up with computerized tomography (CT).

**Figure 2.**
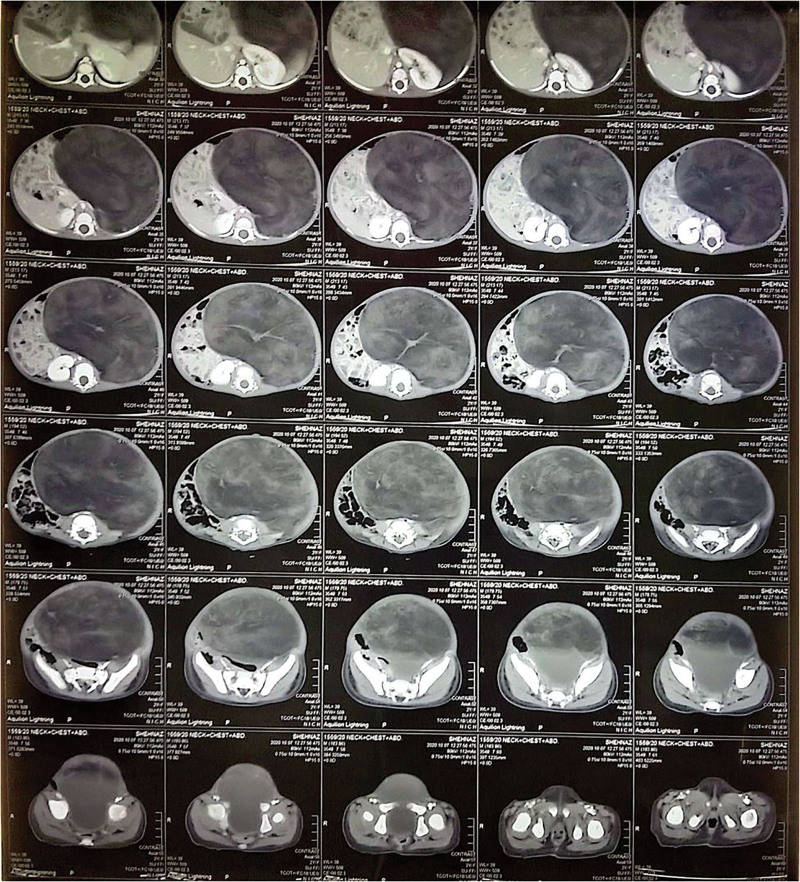
Computed tomography scan of the head, chest, and abdomen showing the retroperitoneal lipoma.

**Figure 3.**
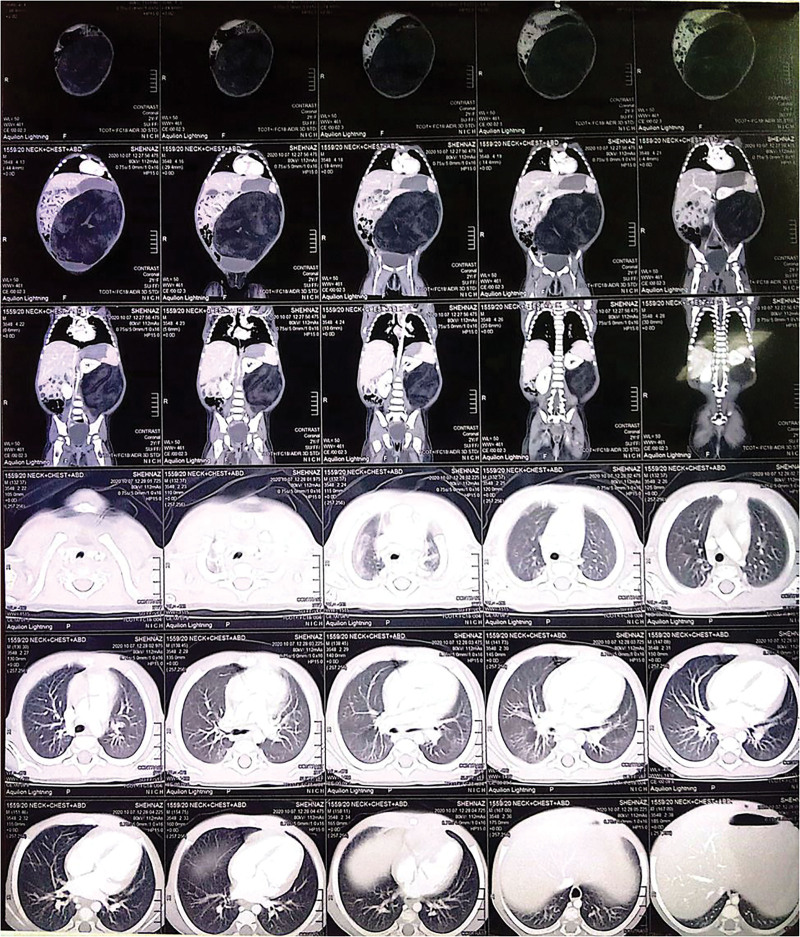
Computed tomography scan of the head, chest, and abdomen showing the retroperitoneal lipoma pushing the left kidney upwards while shifting the intestinal loops towards the right.

**Figure 4.**
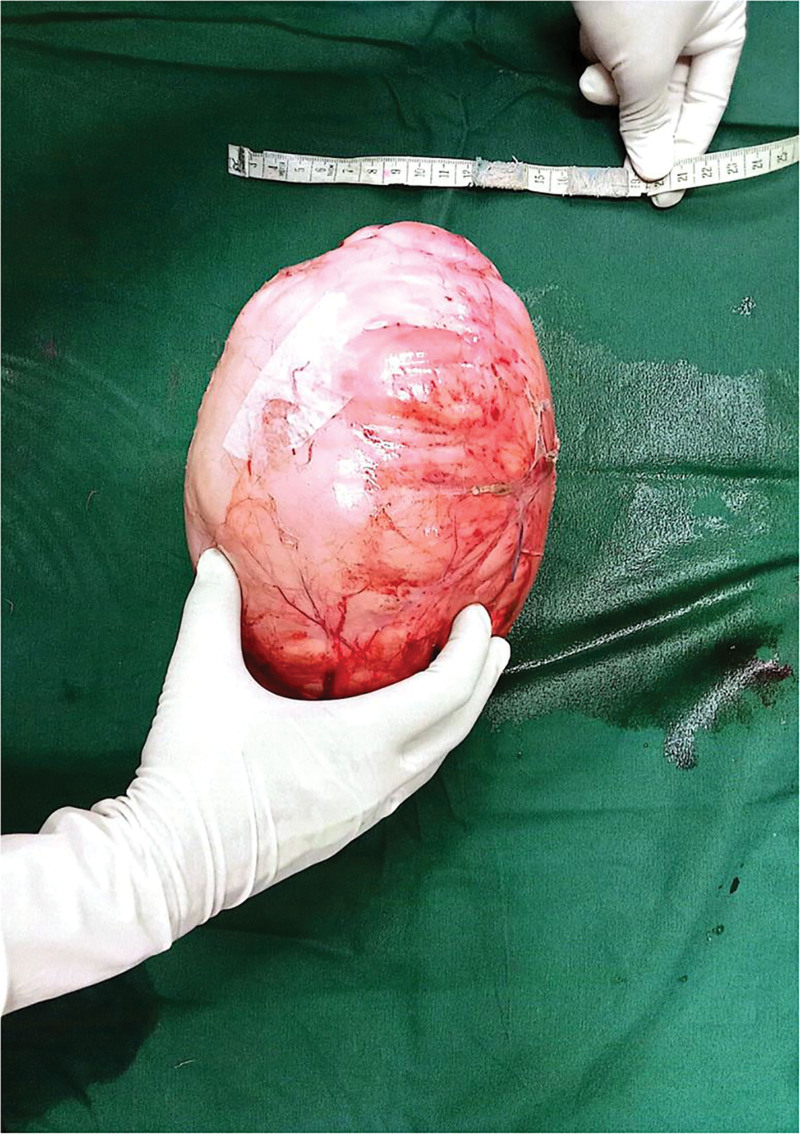
Size of retroperitoneal lipoma of 18.4 × 14.8 × 13.6.

A CT scan with contrast-enhanced of the neck, the thorax, and the abdomen revealed a well-circumscribed, non-enhancing, heterogeneous hypodense place on the left side of the abdomen, from the left hypochondrium to the pelvis (Figs. 2 and 3). The mass measured about 18.4 × 14.8 × 13.6 cm (Fig. 4), and the Hounsfield units are between −66 and −90, which are typical of fatty tissue. There were also internal septations that led to compression and rising of the left kidney and spleen, and the bowel loops moved to the right. There were no calcifications, fat necrosis, or any evidence of malignancy found in the lesion. Organs adjacent to it, such as the liver, pancreas, spleen, kidneys, and bowel structures, were not remarkable.

**Figure 5.**
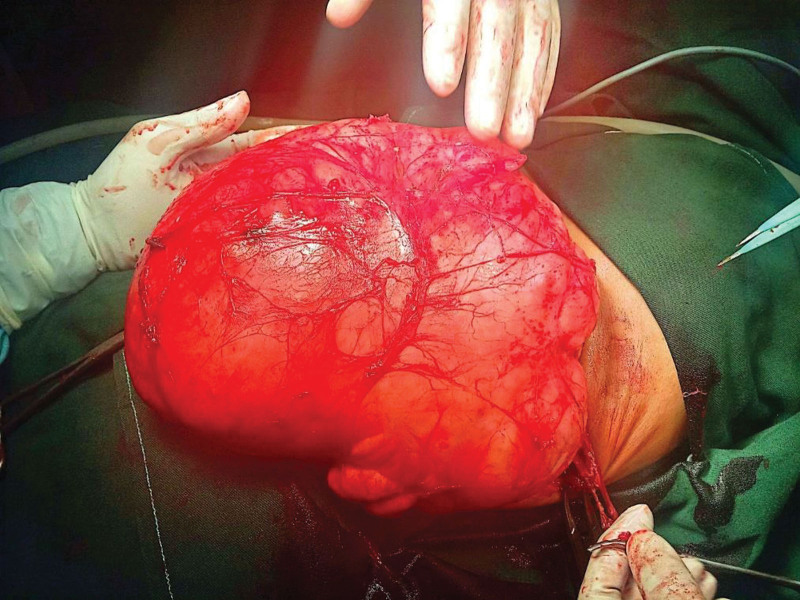
Surgical procedure of retroperitoneal lipoma.

The imaging data were indicative of a benign neoplastic process with lipoma or lipoblastoma as the most likely diagnosis; nevertheless, the malignant transformation could not be excluded. Due to its large size and compressive force, the patient was explored surgically (Fig. 5). A large, encapsulated yellow-tint fatty mass was found intraoperatively in the retroperitoneum, originating below and to the left of the kidney. This was a lobulated cystic lesion of about 2 kg in weight, which was making an upward pull on the left kidney and pushing loops of intestine to the right. The operation was complete and without complication, and a part of the adjacent regional lymph node was also biopsied.

The histopathological analysis of the specimen confirmed the diagnosis of a benign lipoma, characterized by mature adipocytes and thin fibrous septa, without any malignant signs such as atypia or necrosis. The analyzed lymph node of the region showed hyperplasia of a reactionary nature with no tumor invasion. The patient had an uneventful postoperative period, and she was discharged home on the fourth postoperative day. At her 6-week follow-up, the patient remained asymptomatic.

## 3. Discussion

In contrast to subcutaneous lipomas, which are linked to hyperlipidemia, obesity, and trauma, the etiology of the retroperitoneal lipomas is unidentified. Nonetheless, several ideas have been suggested, including hormone therapy, disruptions in glucose metabolism, and seeding after the excision of a fibroid. A study suggests retroperitoneal lipoma in pediatric patients is a unique neoplasm linked to specific chromosomal anomalies.^[6]^

A study reported chromosomal abnormalities have been documented in 476 lipomas, with the 12q13~15 implicated in more than three hundred cases. The recombination at 12q13~15 frequently involves many other chromosomal bands, predominantly 3q27~28, analogous with the translocation t(3;12) (q27~28; q14~15). The primary gene that fuses with HMGA2 in lipoma is lipoma-preferred partner (3q27).^[7]^

Lipoblastoma, a rare neoplasm, arises from embryonic white fat tissue and is characterized by its encapsulating and well-circumscribed nature, akin to lipoma; nevertheless, it often recurs post-excision, in contrast to lipoma, which generally is not recurrent.^[8]^ A distinctive liposarcoma cannot be excluded preoperatively from retroperitoneal lipoma, even in cases where a benign lipoma is systematically indicated. Consequently, intraoperative assessment of tumor features and subsequent decisions regarding the degree of resection are critically significant.^[9]^

Sole extirpation must be limited to well defined tumors. Nevertheless, the substantial size of the tumor complicates preoperative assessments of resectability according to CT scans. Consequently, tumor debulking for symptomatic alleviation may be considered if oncological excision is impracticable. Given the potential malignancy of retroperitoneal tumors, resection needs to be performed by a qualified oncologist with precision.^[10]^

CT scan is very effective for detecting tumors containing adipocytes. A relevant clinical phenomenon is well-circumscribed liposarcomas, which most often consist of well-differentiated thalami with slender septa. Imaging features, such as nodularity suggestive of dedifferentiation, thicker segmentation (2 mm+), and widespread localization of the tumor, are suggestive of malignancy.^[11]^ Although the utilization of computed tomography (CT) has numerous advantages, it also has major limitations to it, one being that it cannot reveal tiny infiltrations or the presence of tiny metastatic foci; this lack is what causes the need to have postoperative radiological observation.^[12]^ Surgery is the only known form of treatment for retroperitoneal lipoma because the condition is not responsive to chemotherapy and radiotherapy.^[13]^ Consistent with our case, seventeen retroperitoneal lipoma cases reported to date, from 1980,^[14–30]^ as shown in Table 2. Only two of them are pediatric cases and the recurrence rate is such that aggressive resection is required, which, although necessary, has a propensity toward morbidity and the difficulties in the management of the disease.

**Table 2:** Compendium of reported cases of retroperitoneal lipomas in humans from 1980. Only 3 cases of the pediatric population are reported.

Case	Year	Gender	Age	Tumor size (in cm)
Deppe et al^[14]^	1985	F	26	11 × 8 × 3
Zhang et al^[15]^	1987	M	65	50 cm (diameter)
Acheson et al^[16]^	1997	F	76	20 × 20 × 12
Matsubara et al^[17]^	2000	M	65	12 × 13
Marshall et al^[18]^	2001	M	47	N/A
Forte et al^[19]^	2002	M	61	N/A
Foa et al^[20]^	2002	M	52	10.5 × 9.5 × 2
Ukita et al^[21]^	2009	F	65	15 cm (diameter)
Chander et al^[22]^	2012	F	36	13.6 × 11.2 × 9.1
Saito^[23]^	2013	M	65	30 cm (diameter)
Hardy and Goliath^[24]^	2015	F	13	19.5 × 16.6 × 8.8
Weniger et al^[25]^	2015	F	73	55 × 10
Singh et al^[26]^	2019	M	05	N/A
Mitchell et al^[27]^	2020	F	29	28 × 14 × 6
Petca et al^[28]^	2022	M	53	36.5 × 21 × 16.5
Wubishet et al^[29]^	2025	M	03	18.5 × 9.7 × 8.3
Kotohata et al^[30]^	2025	F	38	20 × 17 × 7
Our Case	2025	F	02	18.4 × 14.8 × 13.6

## 4. Conclusion

The case propagates the necessity of timely diagnosis of retroperitoneal malignancies, and the much-needed fundamentals of preoperative imaging in the decision-making in surgery. Hormonal therapy and glucose metabolism perturbations have been postulated to be the etiology of the retroperitoneal lipoma. At the same time, iatrogenic seeding after fibroid excision and chromosome translocations might also cause lipoma formation. It also contributes to a scanty literature on pediatric retroperitoneal disease by offering insights into the best strategies for both surgery and diagnosis.

### 4.1. Patient perspective

The patient’s attendants were very satisfied with the surgery and hospital stay. They thanked the duty doctors for their continuous support and successful surgery.

## Author contributions

**Conceptualization**: Batool Fatima.

**Data curation**: Batool Fatima.

**Formal analysis**: Muhammad Umer Farooq Mujahid.

**Methodology**: Neha Rubab, Sakarie Mustafe Hidig.

**Project administration**: Neha Rubab.

**Supervision**: Sakarie Mustafe Hidig.

**Validation**: Neha Rubab.

**Visualization**: Neha Rubab.

**Writing – original draft**: Muhammad Umer Farooq Mujahid.

**Writing – review & editing**: Sakarie Mustafe Hidig.
